# STK33/ERK2 signal pathway contribute the tumorigenesis of colorectal cancer HCT15 cells

**DOI:** 10.1042/BSR20182351

**Published:** 2019-03-01

**Authors:** Shengjun Zhang, Haoyu Wu, Kaiyu Wang, Minli Liu

**Affiliations:** 1 Department of General Surgery, Affiliated Hospital of Yan’an University, Yan’an, Shaanxi 716000, China; 2 Department of Glandular Vascular Surgery, Affiliated Hospital of Yan’an University, Yan’an, Shaanxi 716000, China; 3 Department of Pathology, Medical College of Yan'an University, Yan’an, Shaanxi 716000, China

**Keywords:** colorectal cancer, ERK2, HCT15, STK33, tumorigenesis

## Abstract

Serine/threonine kinase 33 (STK33) is a serine/threonine kinase and participates in many apoptotic process. Herein, we found that the extracellular signal-regulated kinase 2 (ERK2) was a substrate of STK33. STK33 phosphorylated ERK2 and increased the activity of ERK2 and promote the tumorigenesis of colorectal cancer HCT15 cells. Clinical simple showed that STK33 was highly expression in colorectal cells and tissues. *Ex vivo* and *in vivo* studies demonstrated that STK33 accelerate tumorigenic properties in NCM460 cells and athymic nude rats. *In vitro* kinase assay results indicated that STK33 can phosphorylate ERK2. *Ex vivo* studies further showed that STK33 can bind with ERK2 and take part in the regulation of ERKs signaling pathway. In short, our results showed that STK33 is a novel upstream kinase of ERK2. It may provide a better prospect for STK33 based prevention and treatment for colorectal cancer patients.

## Introduction

Globally, colorectal cancer (CRC) is the third malignant tumor in human according to a previous epidemiological survey data [1]. It is also one of the main causes of cancer-associated mortality in the world. Each year, about 1 million new CRC cases was diagnosed and 30% of them who succumb to CRC each year in China [2–4]. Despite developments in diagnostic and therapeutic strategies, which have already ameliorated the survival rates of patients with early stage CRC, but the prognosis of patients with late stage CRC still poor [5,6]. Therefore, further investigations are required to gain an improved understanding of the molecular characteristics and associated biological mechanisms underlying the proliferation, migration, and metastasis of CRC cells. This may also enable the identification of early screening markers and therapeutic targets.

The extra cellular signal-regulated kinases (ERKs) are a member of mitogen-activated kinase (MAPK) which is activated by Ras, Raf, and MEK [7,8]. After activated, ERKs translocates from the cytoplasm to the nucleus and then take part in the gene transcription, cell proliferation, and differentiation [9,10]. ERKs subcellular localization is adjusted by some proteins, such as MEK and TOPK, which is responsible to ERKs activation, and the positive feedback loop between TOPK and ERK2 increases tumorigenesis properties of HCT116 CRC [11].

The human serine/threonine kinase 33 (STK33) enzyme belongs to calcium/calmodulin-dependent kinase family and is located on chromosome 11p15.3, which is a gene-rich region associated with several diseases, including cancer [12]. A previous study demonstrated that STK33 is expressed in a variety of normal tissues but at very low levels after investigating the expression of STK33 mRNA and protein in normal human adult and embryonic tissues [13]. However, it was observed to be highly expressed in the testis, especially in the spermatogenic epithelium. It has also been demonstrated that STK33 involved in the ‘synthetic lethality’ process in a variety of tumor cells, which occurs when deficiency in the expression of multiple genes results in cell death and depends on the Ras oncogene [14]. Previous studies have demonstrated that STK33 may serve a significant role in molecular targeted therapy for KRAS-dependent tumors [14]. Furthermore, STK33 is overexpressed in hypopharyngeal squamous cell carcinoma [15], hepatocellular carcinoma [16], human large cell lung cancer [17], and pancreatic cancer [18] and the increased expression of STK33 may subsequently promote tumorigenesis and disease progression.

However, the detailed mechanisms of STK33 signaling still unknown based on the published data. In our study, we have explored the question whether STK33 can promote cell transformation and detected the specific mechanisms.

## Materials and methods

### Cell culture

The HCT15 CRC cell lines, NCM460 colorectal normal cell lines, and HEK293 cells were ordered from America Type Culture Collection (Manassas, VA, U.S.A.). They were cultured in cell incubator at 37°C in their respective medium with 10% FBS. The cells were treated with 20 ng/ml EGF for 15 min after starvation overnight in no-FBS medium. Stable cell lines were screen by G418.

### Plasmids and siRNA preparation

The pcDNA3-HA-STK33 plasmids were ordered from Addgene (Addgene, U.S.A.). NCM460 stable cell lines transfected with pcDNA3-HA-STK33 plasmid were screened by G418. Hairpin siRNA of STK33 was synthesized by Santa Cruz Biotechnology (Santa Cruz, CA, U.S.A.). The sense sequence for shSTK33 was 5-TGACCCAAGTATCTCTCAGACTCGTTCAACAGAGCACAATTATACCTTGGCGA-3, and the antisense sequence was 5-CGCTTTACCAAATTTGAGGTTTGTAAAATTCTCTTTTCTTGAAGCCTTTCG-3. Then, they were cloned into the pSilencer 3.1-H1 neo vector following the protocal. The shSTK33 and ShMock plasmids were transfected into HCT15 cells, and the stable cell lines were screened by G418. NpT7-5ERK1 and NpT7-5ERK2 were purchased from Santa Cruz Biotechnology (Santa Cruz, CA, U.S.A.). The recombinant proteins His-ERK1 and His-ERK2 were purified using Ni-NTA agarose (Qiagen, Valencia, CA, U.S.A.), and GST-ERK2 was purified using GST-TAG agarose (Qiagen, Valencia, CA, U.S.A.) obtained.

### Antibodies and reagents

Anti-STK33, anti-ERKs, anti-p-ERKs, anti-p-ERKs, anti-p-CREB, anti-p-c-FOX, anti-p-ELK1, and β-actin were purchased from Cell Signaling Technology (Danvers, MA, U.S.A.). The anti-HA-probe and anti-V5-probe were from Proteintech Group, Inc (Chicago, U.S.A.). Active STK33 was ordered from Millipore (Billerica, MA, U.S.A).

### Immunohistochemical

After the mice were euthanized, the tumors were fixed in 4% formalin, routinely processed, and embedded in paraffin. Sections of 5 μm were placed on glass slides for H&E staining and IHC analysis. Tissue array of 22 CRC cell lines were ordered from IMGENEX (San Diego, CA, U.S.A.). The sections and tissue array were treated with 3% H_2_O_2_ for 10 min and blocked with 5% goat serum for 30 min at room temperature. Next, they were incubated at 4°C for overnight with STK33 antibodies (1:200). Then they were washed in PBS and incubated with the secondary antibody respectively (1:500) for 30 min. After PBS washing, the sections were incubated with 3, 3′-diaminobenzidine (DAB) as substrate for 3 min. Images were obtained and 400× magnified using an Olympus Imaging System Microscope.

### Immunoprecipitation and Western blotting

Different cell lines (7 × 10^5^) were cultured in 10-cm-diameter dishes, and the cells were harvested and disrupted in 200 µl of RIPA buffer. The lysates were sonicated 15 s for three times and centrifuged at 14,000 rpm for 10 min. The quantity of protein was detected by the BCA method. The samples with 5× SDS loading buffer were heated at 98°C for 5 min, and then cooled on ice. Next, the samples were separated on a 10% SDS-PAGE and subsequently transferred onto a PVDF membrane. The proteins were determined by chemiluminescence. All antibodies were used in Western blot analysis (1:1000 dilutions) by following the protocal of the manufacturers for immunoprecipitation (IP). The transfected HEK293 cells were harvested in 1% CHAPS buffer. The protein (100 mg) was subjected to IP following the manufacturer’s protocol.

### *In vitro* kinase assay

The STK33 active kinase and kinase buffer (10×) were ordered from Millipore Corp. (Billerica, MA, U.S.A.). The inactive ERK1 and ERK2 substrate were incubated at 30°C for 30 min in 1× kinase buffer containing 200 µmol/l ATPor1 μCi [γ-32P] ATP. The samples were analyzed by autoradiography or Western blot.

### Anchorage-independent transformation assay

The cell lines (8 × 10^3^/well) were cultured in six-well plate in 1 ml of 0.3% BME (Eagle basal medium) agar containing 10% FBS, 25 μg/ml gentamicin and 2 mM L-glutamine. The cultures were maintained in a 37°C, 5% CO_2_ incubator for 10 days. The cell’s colonies were scored by using a Motic Image Plus computer program.

### Confocal laser scanning fluorescence microscopy

HCT15 cells were fixed in methanol (−20°C). The cells were incubated overnight with the STK33 antibodies at 4°C after blocking in 5% normal goat serum at room temperature for 1 h. Then, the cells were incubated at room temperature for 1 h with the Alexa Fluor 488 (green for STK33) or Alexa Fluor 546 (red for ERKs) conjugated secondary antibody. Colocalization of proteins was observed by Leica SP8 Confocal Microscope (Leica Microsystems Inc., Germany).

### *In vivo* xenograft mouse model

Athymic Balb/c nude mice (4–6-week-old males) were ordered from Shanghai Boao Bioscience Co., Ltd (Shanghai, China). The animals were performed using protocols approved by Research Animal Resources, Laboratory Animal Center, The Fourth Military Medical University (China). Each kinds of the cell lines (5 × 10^5^ in 200 µl PBS) was injected subcutaneously into the left flank of the mice, and tumor volume was detected based on the following formula: tumor volume (mm^3^) = (length × width × height × 0.52). The mice were killed until tumors reached 1 cm^3^ total volume. The tumors were dissected and sent for immunohistochemical analysis.

### Immunohistochemical analyses for tissue

Following the manufacturer’s protocol, immunohistochemistry was performed on paraffin-embedded array specimens or specimens of samples from athymic nude mice using the VECTASTAIN ABC Kit (Vector Laboratories, Burlingame, CA, U.S.A.). A STK33 antibody was used (1:100) in the immunohistochemical analyses.

### Ethics statement

The study protocol was approved by the Institutional Review Board of Affiliated Hospital of Yan’an University. Informed consent was confirmed by the Institutional Review Board (permit number: 2017-001). The animal studies were performed after receiving approval of the Institutional Animal Care and Use Committee of Fourth Military Medical University (IACUC approval no. 2017-024). All efforts were made to minimize suffering.

### Statistical analysis

Statistical analysis was performed using Graphpad prism. Student’s *t* test was used to evaluate the data. In all tests, differences were considered significant at *P*<*0.05*.

## Results

### STK33 is overexpressed in human CRCs and CRC cell lines

**Figure 1:**
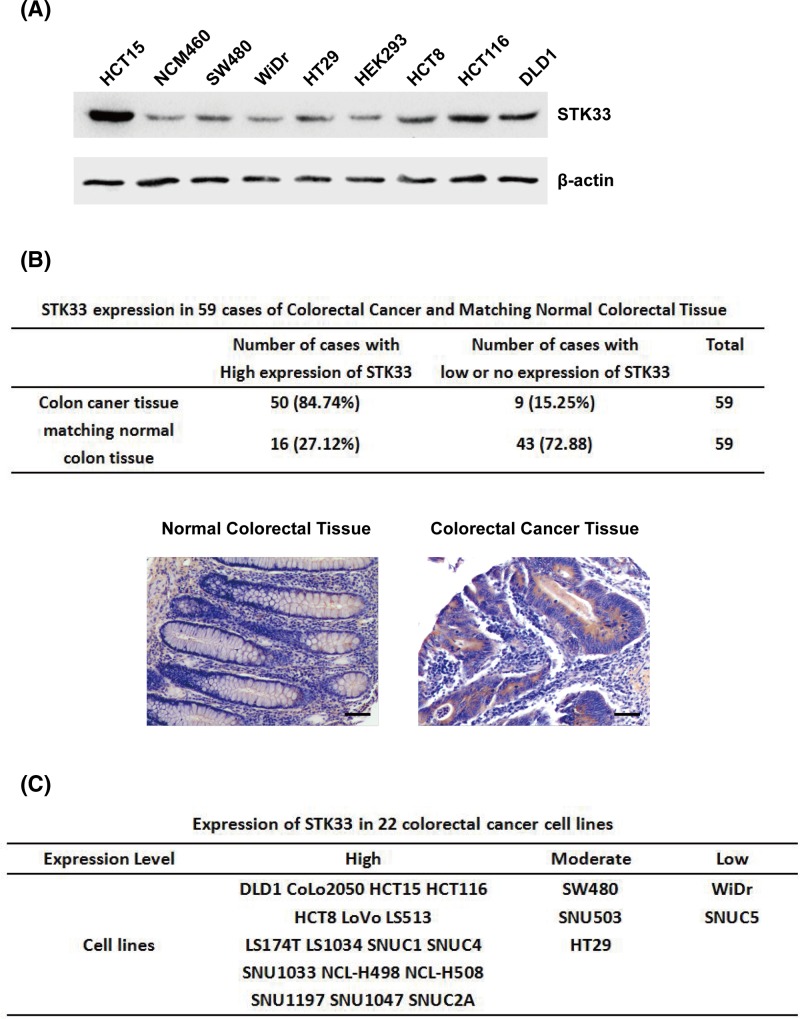
STK33 is overexpressed in CRC cell lines and CRC patients (**A**) STK33 expression in nine different cell lines. (**B**) Immunohistochemical detection for STK33 expression in normal colorectal tissue and matching 59 cases of human CRC tissues. The scale bars from each group is 50 μm. (**C**) The expression of STK33 in different CRC cell lines.

STK33 overexpression has been observed to take part in angiogenic program in hypoxic tumors [19]. To detect the expression of STK33 in human CRC cell lines and normal cell lines cancer cells, seven different CRC cell lines and two noncancer cell lines were detected by Western blotting. The results showed that STK33 was overexpressed in human colorectal carcinoma HCT15, HCT116, HCT8, and DLD1 cells. What is more, STK33 was moderately expressed in human colorectal carcinoma SW480 and HT29 cells. Furthermore, STK33 was low expressed in human normal colorectal cell lines NCM460 cell and human embryonic kidney 293 cells (Figure 1A).

The expression STK33 was further detected in human CRC tissues. On the one hand, STK33 expression was detected by immunohistochemistry tissue array analyses in normal colorectal tissue and matching CRC tissue samples from 59 cancer patients (IMGENEX). The results showed that STK33 was highly expressed in 50 (84.74%) of the 59 colorectal cancerous tissues but in only 16 (27.12%) of the 59 matching normal colorectal tissues (Figure 1B, table). The immunohistochemistry staining statistical data of STK33 expression are shown in Figure 1B. The cytoplasm of CRC tissues was more brown than that of normal group, which demonstrated STK33 was higher expression in CRC tissues (Figure 1B). On the other hand, STK33 expression was detected in 22 CRC cell lines by immunohistochemistry using a commercially available array (IMGENEX). The results showed that STK33 were highly expressed in 17 CRC cell lines, moderately expressed in three cancer cell lines and only low expressed in two cancer cell lines, respectively (Figure 1C). The HCT15 cell line was used in the remaining experiments because it was highly expressed STK33. The above results verified that STK33 is highly expressed in human CRCs tissues and CRC cell lines.

### STK33 promotes NCM460 cells transformation *in vivo* and *in vitro*

**Figure 2:**
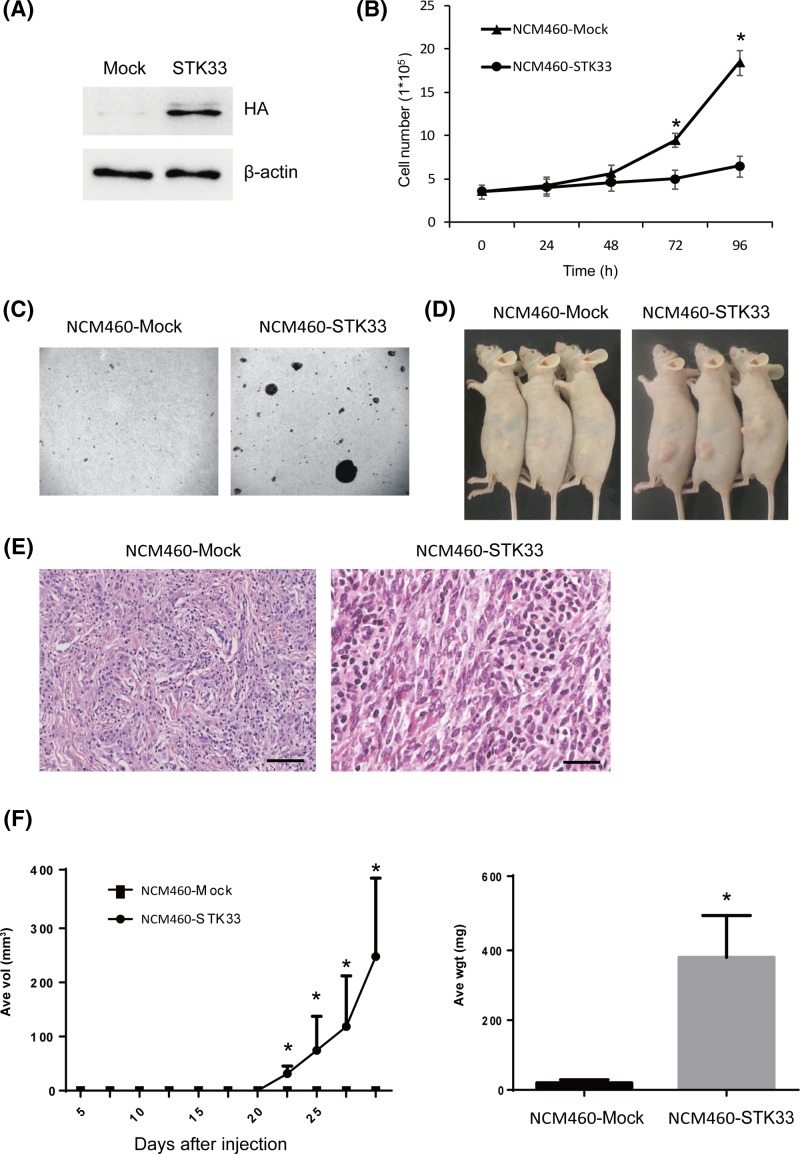
STK33 promotes the transformation of normal human colorectal NCM460 cells *in vivo* and *in vitro* (**A**) The STK33-overexpressing cells (NCM460-STK33) and vector control cells (NCM460-Mock) were detected by Western blotting. (**B**) Growth curves of these cells. (**C**) STK33 can promote NCM460 cells transformation *in vitro* by the independent anchored transformation assay. (**D**) STK33 can promote the transformation of NCM460 cells *in vivo*. (**E**) Representative H&E staining obtained from the NCM460-STK33 group. The scale bars from left to right correspond to 250 and 25 μm, respectively. (**F**) Tumor growth curve and average tumor weight of mice injected with NCM460-STK33 or NCM460-Mock cells. The asterisk indicates a significant increase (P<0.01) in NCM460-STK33 group compared with NCM460-Mock group.

STK33 was highly expression in cancerous tissues, whereas, it was found very low expression in normal tissues [13]. To answer the question whether STK33 is oncogenic associated with malignant transformation or is just one of many up-regulated proteins. The above study has showed that NCM460 cells expressed very low levels of endogenous STK33 (Figure 1A). So we detected whether STK33 can transform NCM460 cell line. After pcDNA3-HA-STK33 vector (NCM460-STK33) or pc-DNA3 (NCM460-Mock) were overexpressed in NCM460 stable cell lines (Figure 2A), the growth curves of NCM460-Mock and NCM460-STK33 cells were compared and the results showed that NCM460-STK33 cell lines growth faster than NCM460-Mock cell lines (Figure 2B). Furthermore, the colony formation of NCM460-Mock and NCM460-STK33 cells was contrasted through the independent anchored transformation assay. The results demonstrated that NCM460-STK33 cells colonies were more and larger than NCM460-mock cells, indicating a greater malignant transformation potential of NCM460-STK33 cells (Figure 2C). The above studies indicated that overexpression of STK33 is strongly related to malignant cell transformation *in vitro*.

Then, we examined whether overexpression of STK33 could lead to neoplastic transformation athymic nude mice. The results showed that mice injected with NCM460-Mock cells developed a few tumors, whereas the mice inoculated with NCM460-STK33 cells developed very bigger tumors over a 4-week period (Figure 2D). Typical tumors from NCM460-STK33-injected mice were detected by H&E staining. The results indicated a high nuclear/cytoplasmic ratio, abundant mitosis, numerous blood vessels, and marked nuclei pleomorphism in some areas (Figure 2E). These morphological manifestations accord with a diagnosis of high-grade malignant carcinoma. The tumors in NCM460-STK33-injected mice first appeared at 17 days, and the final average tumor weights and tumor growth curves showed that STK33 overexpression promotes NCM460 cells tumorigenic *in vivo* (Figure 2F).

### Knockdown of STK33 in HCT15 CRC cells reduces tumorigenic properties *in vivo* and *in vitro*

**Figure 3:**
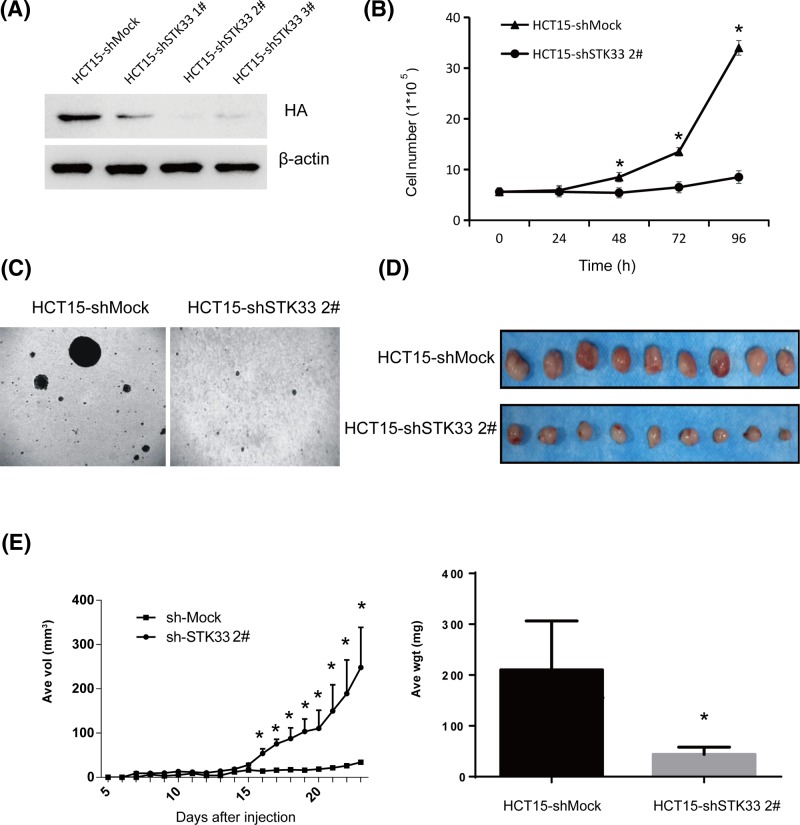
Knockdown of STK33 in HCT15 reduces tumorigenesis *in vivo* and *in vitro* (**A**) The vector control cells (HCT15-shMock) and knockdown STK33 cells (HCT15-shSTK33) were detected by Western blotting. (**B**) Growth curves of these cells. (**C**) Knockdown of STK33 reduces tumorigenesis of CRC cell line HCT15 *in vitro* by the independent anchored transformation assay. (**D**) Knockdown of STK33 reduces tumorigenesis of CRC cell line HCT15 *in vivo*. (**E**) The tumor growth curve and average tumor weight of mice injected with HCT15-shMock or HCT15-shSTK33 cells. The asterisk indicates a significant decrease in tumor volume and weight in HCT15-shSTK33-injected mice compared with HCT15-shMock-injected mice (*P*<0.001).

The above results showed that STK33 was overexpressed in CRC tissues and cell lines. What is more, overexpression of STK33 could transform the normal colorectal NCM460 cell lines into a tumorigenic cell line. In contrast, if STK33 expression was suppressed by knocking down STK33 in cancer cell lines which expresses high levels of STK33, we want to know whether tumorigenic properties can be reduced. So, an HCT15-shSTK33 cell line was generated by stable transfection of pSilencer 3.1-H1-STK33-shRNA into HCT15 cells. The HCT15-shMock cell line was made as the control group (Figure 3A). Growth curves results showed that HCT15-shSTK33 cells grew much slower compared with HCT15-shMock cells (Figure 3B). Next, the colonies formed ability of HCT15-shMock and HCT15-shSTK33 cells was detected by independent anchored transformation analysis. The results demonstrated that the anchorage-independent growth ability from HCT15-shSTK33 cells were much smaller than HCT15-shMock cells (Figure 3C). These above results indicated that inhibition of STK33 can suppress the tumorigenesis of HCT15 cell lines *in vitro*.

Next, we wanted to know whether using siRNA against STK33 (shSTK33) in HCT15 cells could suppress tumors in athymic nude mice. HCT15-shMock or HCT15-shSTK33 cells (4 × 10^6^) were injected subcutaneously into the left flank of the 7-week old mice. More than two-third mice from two groups developed tumors, therein, the tumors in HCT15-shSTK33-injected mice were much smaller than that in the HCT15-shMock-injected mice (Figure 3D). The final average tumor weight and volume in HCT15-shMock inoculated mice were remarkable larger than in the mice injected with HCT15-shSTK33 cells (Figure 3E). These results further indicated that inhibiting STK33 expression in HCT15 cells can significantly reduce the tumorigenesis of HCT15 CRC cells *in vivo*.

### STK33 phosphorylates ERK2 *in vitro*

**Figure 4:**
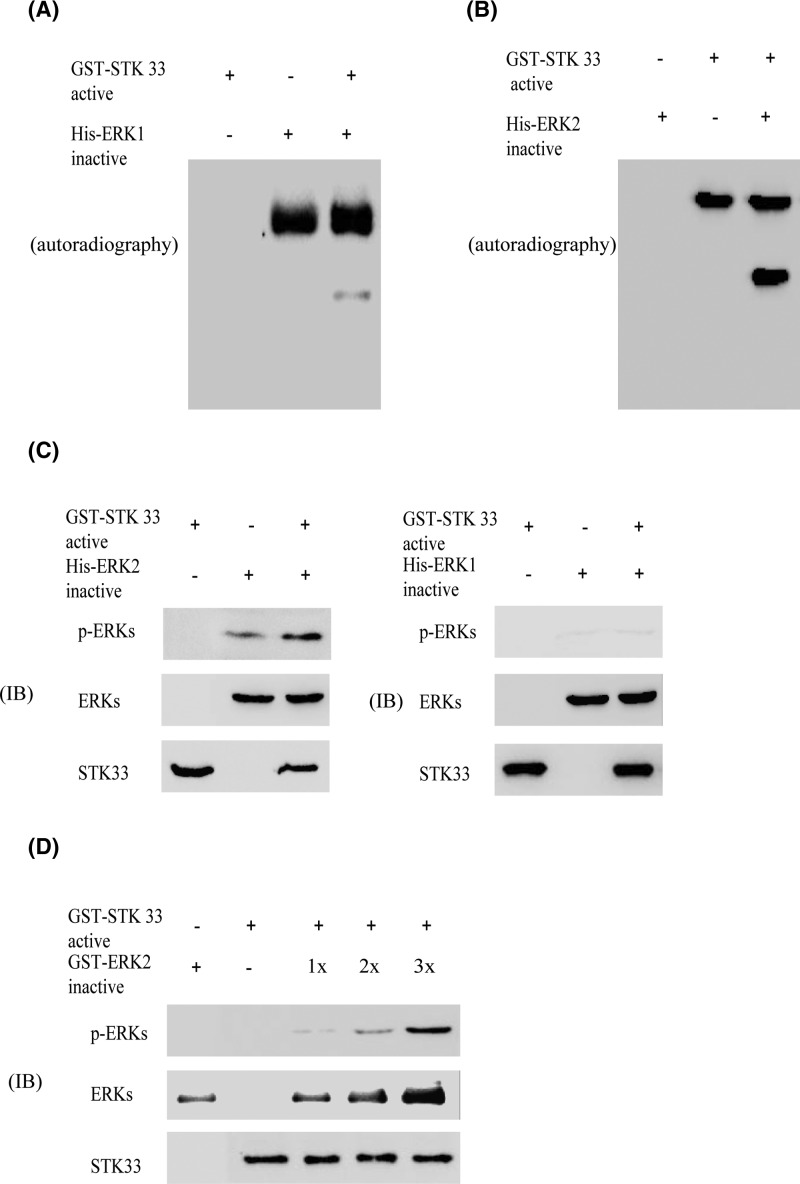
STK33 phosphorylates ERK2 *in vitro* (**A**) Active STK33 phosphorylates inactive His-ERK1 in the presence of [γ-32P]-ATP as visualized by autoradiography *in vitro*. (**B**) Active STK33 phosphorylates inactive His-ERK2 in the presence of [γ-32P]-ATP as visualized by autoradiography *in vitro*. (**C**) Active STK33 phosphorylates inactive ERK2 *in vitro*. (**D**) The phosphorylation level of STK33 increases in a dose-dependent manner corresponding to the increased amount of inactive ERK2.

We have known that STK33 has strong transformation potential. However, the specific signaling pathway of STK33 is still unclear. ERKs are one of the members of MAPK family and activated ERKs induce many cellular events, including cell survival and proliferation. Now ERKs is the mainly known downstream target of MEK. So, we try to uncover the new kinase which relationship with ERKs. First, we detected whether STK33 can phosphorylate inactive ERK1 or ERK2 in the presence of [γ-^32^p]-ATP *in vitro*. His-ERK1 and His-ERK2 were used as the substrate of active STK33. The results indicated that active STK33 could very weakly phosphorylate ERK1 (Figure 4A), but might strongly phosphorylate ERK2 (Figure 4B).

Furthermore, we detected the results of the *in vitro* kinase assay through Western blotting. Results showed that STK33 was almost no phosphorylate ERK1, but the phosphorylation of ERK2 was significantly detected (Figure 4C). Furthermore, in the *in vitro* kinase assay experiment, the different dose of inactive ERK2 was used as the substrate of active STK33. Results revealed that ERK2 can be phosphorylated in a dose-dependent manner by active STK33 (Figure 4D). The above results showed that STK33 can phosphorylate ERK2 *in vitro*.

### STK33 phosphorylates ERK2 *ex vitro*

**Figure 5:**
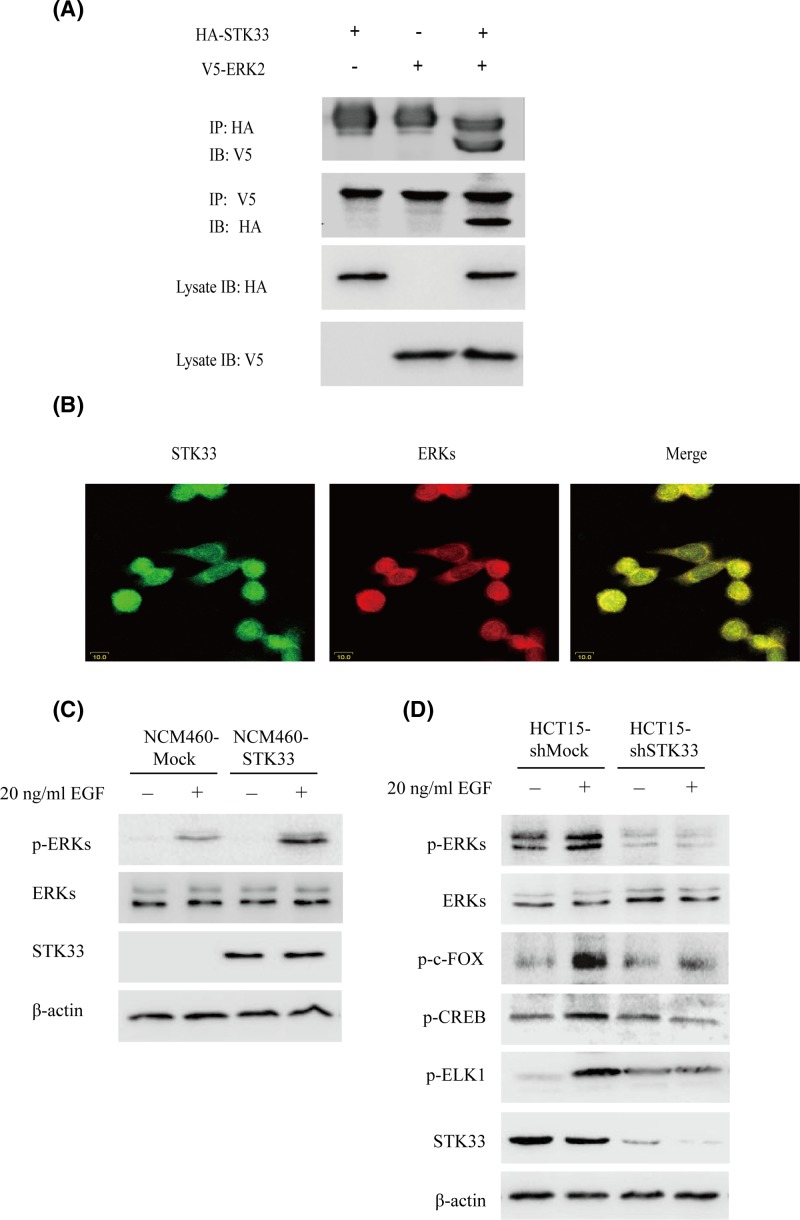
STK33 phosphorylates ERK2 *ex vitro* (**A**) STK33 binds with ERK2 in 293T cells pcDNA3-HA-STK33 and pcDNA3-V5-ERK2 were cotransfected into 293T cells by transient transfection, immunoprecipitated with a HA or V5 antibody, and then probed with V5 or HA, respectively. (**B**) Colocalization of STK33 and ERKs was observed by confocal microscope in HCT15 cells. Nuclear and cytoplasmic staining of STK33 and ERKs was mostly merged together. (**C**) The phosphorylate level of ERKs was increased in NCM460-STK33 cells after EGF treatment for 15 min. (**D**) The downstream signaling pathways of ERKs are detected by Western blotting in HCT15-shSTK33 cells or HCT15-shMock cells.

From the above data, we speculated that there is the apparent interaction between STK33 and ERK2 *ex vitro*. First, we detect whether ERKs can bind with STK33 by IP experiment. The experiment results showed that STK33 could bind with ERK2 after overexpression in HEK293 cells (Figure 5A). Furthermore, endogenous STK33 and ERK2 were detected by confocal microscope in HCT15 cells. This result also showed that STK33 (green) colocalized with ERKs (red) in both the cytoplasm and nucleus HCT15 cells (Figure 5B). As our data have showed that STK33 could transform NCM460 cells *in vivo* and *in vitro*, we want to know the expression of phospho-ERKs in NCM460-Mock and NCM460-STK33stable cell lines under the condition of EGF stimulation. The results demonstrated that the phosphorylation level of ERKs in NCM460-STK33 cells were higher than NCM460-Mock cells (Figure 5C). Correspondingly, the phosphorylation of ERKs was detected in HCT15-shMock, HCT15-shSTK33 cells after 20 ng/ml EGF stimulating 15 min. Results showed that the expression of phospho-ERKs sharply decreased after STK33 was blocked by shSTK33 (Figure 5D).

Furthermore, we detected the downstream signaling pathway of ERKs when STK33 was blocked by shSTK33. Results demonstrated that the expression of phospho-c-FOS, phospho-CREB, and phospho-ELK1 were decreased in HCT15-shSTK33 cells compared with STK33-shMock, which further confirming that STK33 can regulate ERKs signaling (Figure 5D).

## Discussion

STK33 is a novel serine/threonine kinase that has been the focus of cancer studies in recent years. Previous studies have demonstrated that STK33 is overexpressed in hypopharyngeal squamous cell carcinoma [20], hepatocellular carcinoma [21], human lung cancer [16,22,23], and pancreatic cancer [18] and the increased expression of STK33 may subsequently promote tumorigenesis and disease progression. Furthermore, Yin et al. studies indicated that STK33 was hypermethylated in CRC cell lines and promoted the proliferation of CRC cells [24]. However, the specific STK33 signaling pathway in promoting CRC tansformation and proliferation is not clear.

In our study, we found that STK33 promotes the transformation of colorectal carcinoma. Furthermore, STK33 was highly expressed in CRC tissues, but was lowly expressed in normal tissue. Knocking down STK33 in HCT15 CRC cells suppressed tumorigenesis and overexpression of STK33 could promote normal colorectal NCM460 cells transformation *in vitro* and *in vivo*. Surprisingly, STK33 could contribute to the STK33-induced transformation through phosphorylating ERK2. Inhibiting STK33 by shSTK33 lead to a down-regulation of phosphorylation of downstream substrates of ERKs such as ELK1, CREB or c-FOS. These results also suggested that STK33 could be a promising drug target for tumor chemotherapy because STK33 expression seems not in normal cells except in the testis and mainly in cancer cells [12,13].

Ras/Raf/MEK/ERKs pathway plays an important role during the development of tumor. Previous studies showed that STK33 may serve a significant role in molecular targeted therapy for KRAS-dependent tumors [14]. By contrast, a different study demonstrated that the activity of STK33 may be nonessential in KRAS-dependent cell lines [25]. Therefore, many questions related to STK33 in tumor cells remains controversial and the mechanisms underlying the function of STK33 in tumor biology are complex. Therefore, more research is required to further elucidate the mechanism of how STK33 promotes CRC progression.

## Abbreviations

CRC: colorectal cancer
CREB: cAMP-response element binding protein
DAB: 3, 3-diaminobenzidine
ERK2: extracellular signal-regulated kinase 2
IHC: immunohistochemical
IP: immunoprecipitation
MAPK: mitogen-activated kinase
STK33: serine/threonine kinase 33
